# Supplementary material for: Developmental hematopoietic stem cell variation explains clonal hematopoiesis later in life
Source: Nat Commun. 2024 Nov 26;15:10268. doi: 10.1038/s41467-024-54711-2 (PMC11599844; doi:10.1038/s41467-024-54711-2)
Supplement: Supplementary file 4 — Source Data [file 41467_2024_54711_MOESM4_ESM.zip › Files in ZIP folder.docx]

TmobT.csv

list of all neutral fCpG sites used in this

studydata.xlsx

Excel spreadsheet listing all the individual data points (Pearson correlation coefficients between

average methylation profile of two individuals) shown in Fig 1F
